# Impact of early corticosteroid therapy on short-term pulmonary function and clinical outcomes in children with severe *Mycoplasma pneumoniae* pneumonia

**DOI:** 10.3389/fped.2026.1867429

**Published:** 2026-08-05

**Authors:** Mingyu Feng, Ping Wei, Li Wang, Tianhui Chen, Liya Yang

**Affiliations:** 1 Department of Pediatrics, Chengdu Shuangliu Hospital of Traditional Chinese Medicine, Chengdu, Sichuan, China; 2 Department of Pediatrics, Yuxi Zhongshan Hospital, Yuxi, Yunnan, China; 3 Department of Pediatrics, Lincang People’s Hospital, Lincang, Yunnan, China

**Keywords:** children, corticosteroids, inflammation, *Mycoplasma pneumoniae* pneumonia, pulmonary function

## Abstract

**Background:**

Severe *Mycoplasma pneumoniae* pneumonia (SMPP) in children can lead to pulmonary function impairment. This study aimed to evaluate the impact of early corticosteroid therapy combined with antibiotics versus antibiotics alone on short-term pulmonary function and clinical outcomes in children diagnosed with SMPP.

**Methods:**

A retrospective analysis was performed on 259 pediatric inpatients diagnosed with SMPP at our hospital between October 2022 and June 2025. Patients were divided into the antibiotics alone group (*n* = 158) and the early corticosteroid use group (*n* = 101). Pulmonary function, such as peak expiratory flow (PEF), forced expiratory volume in the first second (FEV1), forced vital capacity (FVC), and small airway function parameters were measured before and after treatment. We also assessed injury markers—including lactate dehydrogenase (LDH), aspartate aminotransferase (AST), and alanine aminotransferase (ALT)—as well as inflammatory cytokines (C-reactive protein [CRP], interleukin-6 [IL-6], and interferon-gamma [IFN-γ]) and immunoglobulins (IgA, IgG, IgM). Clinical symptom resolution times and adverse reactions were compared.

**Results:**

After treatment, the early corticosteroid group showed significantly higher PEF (5.74 ± 0.57 vs. 5.13 ± 0.65 L/s), FEV1 (2.81 ± 0.35 vs. 2.38 ± 0.33 L), and FVC (3.22 ± 0.44 vs. 2.75 ± 0.41 L), along with better small airway function compared to the antibiotics alone group (all *P* < 0.05). The corticosteroid group also exhibited notably lower levels of LDH (398.62 ± 105.43 vs. 492.36 ± 118.74 U/L), CRP (7.62 ± 2.14 vs. 12.35 ± 3.28 mg/L), and IL-6 (14.28 ± 4.53 vs. 24.51 ± 6.75 pg/mL), and higher immunoglobulin levels (all *P* < 0.05). Times to cough disappearance, defervescence, and hospitalization were notably shorter in the corticosteroid group (all *P* < 0.05).

**Conclusion:**

Early corticosteroid use combined with antibiotics was associated with significantly greater short-term improvement in pulmonary function, reduced inflammatory markers, and faster clinical symptom resolution in children with SMPP.

## 1 Introduction

*Mycoplasma pneumoniae* (MP) is a prevalent pathogen causing community-acquired pneumonia in children and adolescents, accounting for approximately 10%–40% of cases depending on age and epidemic cycles (1). While *Mycoplasma pneumoniae* pneumonia (MPP) is often self-limiting and mild, a subset of affected children develops severe *Mycoplasma pneumoniae* pneumonia (SMPP) characterized by extensive pulmonary involvement, prolonged fever, and systemic inflammatory responses (2). The clinical course of SMPP may be complicated by respiratory distress, pleural effusion, and extrapulmonary manifestations, leading to substantial morbidity and prolonged hospitalization (3).

The pathogenesis of SMPP involves not only direct pathogen-mediated injury but also excessive host immune responses. MP infection triggers robust production of pro-inflammatory cytokines, including interleukin-6 (IL-6), tumor necrosis factor-alpha (TNF-α), and interferon-gamma (IFN-γ), all of which contribute to pulmonary inflammation and tissue injury (4, 5). This hyperinflammatory state has been associated with airway obstruction and remodeling, potentially resulting in pulmonary function impairment even after pathogen clearance (6). Studies have demonstrated that children with SMPP may exhibit persistent abnormalities in both large and small airway function, including reduced forced expiratory volume in the first second (FEV1) and peak expiratory flow (PEF), highlighting the necessity for therapeutic approaches that target both infection and inflammation (7).

Macrolide antibiotics, particularly azithromycin, remain the first-line treatment for MPP in children due to their antimicrobial activity against M. pneumoniae and additional immunomodulatory properties (8). However, increasing macrolide resistance worldwide and the inability of antibiotics alone to adequately control the hyperinflammatory response in severe cases have prompted investigation into adjunctive therapies (9). Corticosteroids, with their potent anti-inflammatory effects, have emerged as a logical therapeutic option for SMPP, as they can suppress excessive cytokine production and mitigate immune-mediated lung injury (10).

Current clinical guidelines recommend consideration of corticosteroids for children with SMPP who do not respond adequately to antibiotic therapy alone (11). Several studies have reported that corticosteroid use in SMPP is associated with faster clinical improvement, including earlier defervescence and faster resolution of radiographic abnormalities (12). However, the evidence regarding the impact of early corticosteroid intervention on short-term pulmonary function improvement remains limited (13). Given that pulmonary function impairment following SMPP may persist for months or even years, determining whether early corticosteroid therapy is associated with improved functional recovery is of considerable clinical importance (14). Thus, this retrospective study sought to evaluate the association between early corticosteroid use (combined with antibiotics) and short-term pulmonary function, inflammatory responses, and clinical outcomes in children with SMPP, compared to antibiotics alone.

## 2 Materials and methods

### 2.1 Patient selection

We performed a retrospective study of 259 pediatric inpatients diagnosed with SMPP at our hospital between October 2022 and June 2025. The inclusion criteria were: ① Patients aged 5 to 14 years; ② Meeting the diagnostic criteria for SMPP as per the “Evidence-Based Guideline for the Diagnosis and Treatment of *Mycoplasma Pneumoniae* Pneumonia in Children” (15); ③ Complete clinical data. Exclusion criteria included: ① Use of corticosteroid drugs within the past two weeks; ② Allergy to methylprednisolone or azithromycin; ③ Comorbidities including pulmonary tuberculosis, immunodeficiency, malignancy, or severe heart, liver, or kidney dysfunction; ④ Co-infection with other identified pathogens; ⑤ Use of macrolides or other antibiotics within the past week.

A total of 300 pediatric inpatients were initially screened. Based on the inclusion and exclusion criteria, 41 patients were excluded: 10 due to age outside the 5–14 years range, 8 due to corticosteroid use within the past two weeks, 15 due to co-infection with bacteria or viruses, 7 due to comorbidities (immunodeficiency or malignancy), and 1 due to incomplete medical records. Finally, 259 patients were enrolled in the analysis.

Based on the treatment received after admission, the 259 patients were assigned to the antibiotics alone group (*n* = 158) and the early corticosteroid use group (*n* = 101). The antibiotics alone group received standard antibiotic therapy only, whereas the early corticosteroid use group received adjunctive corticosteroid therapy within 24 hours of admission in addition to antibiotics. This study was approved by the Institutional Review Board (IRB) of Chengdu Shuangliu Hospital of Traditional Chinese Medicine (Approval Number: zyy-ky2026010). Given the retrospective design and the anonymity of the data, the IRB waived the requirement for informed consent.

### 2.2 Diagnostic criteria

In this study, the diagnostic criteria for pediatric MPP patients were as follows (15): (1) Clinical manifestations: presence of respiratory infection symptoms such as fever and cough; (2) Radiological changes: chest x-ray or CT scan indicating pulmonary inflammatory changes; (3) Etiological examination (meeting any one of the following): ① A single serum MP antibody titer ≥1:160 (particle agglutination method); a four-fold or greater increase in MP antibody titers in paired sera during the course of illness; ② Positive detection of MP-DNA or MP-RNA by nucleic acid testing. Patients meeting the above MPP diagnostic criteria and exhibiting any one of the following were defined as having SMPP (16): (1) Significantly increased respiratory rate; (2) Presence of respiratory distress or cyanosis; (3) Radiological evidence of multi-lobar involvement or involvement of ≥2/3 of a single lobe; (4) Occurrence of extrapulmonary complications; (5) Presence of pleural effusion; (6) Room air pulse oxygen saturation (SaO₂) ≤92%.

Of the 259 included patients, 112 (43.24%) were diagnosed by serology alone, 68 (26.25%) by PCR alone, and 79 (30.50%) by both methods. Paired serum samples were obtained in 47 patients (18.15%), of whom 32 (68.09%) demonstrated a fourfold or greater rise in antibody titers. Macrolide resistance was assessed in a subset of patients.

### 2.3 Treatment method

All patients received standard treatment, including maintenance of electrolyte and acid-base balance, physical cooling, expectoration, bronchodilation, and oxygen therapy. The antibiotics alone Group was treated solely with azithromycin: azithromycin (Approval No. H20067075, Hainan Beite Pharmaceutical Co., Ltd., Hainan Province) at a dose of 10 mg/kg/day intravenously once daily for 3 consecutive days, followed by a 4-day break, totaling 7 days (one course of treatment). The early corticosteroid use group, in addition to the antibiotic treatment, received intravenous methylprednisolone sodium succinate (Approval No. H20103047, Jin Yao Heping Pharmaceutical Co., Ltd., Tianjin) within 24 hours of admission. The initial dose was 2 mg/kg/day divided into two administrations for 5 consecutive days; on days 6-7, the dose was reduced to 1 mg/kg/day, administered 1-2 times daily. The total corticosteroid treatment duration was 7 days.

The decision to administer early corticosteroids was based on the treating physician's assessment of disease severity upon admission, in accordance with the “Evidence-Based Guideline for the Diagnosis and Treatment of *Mycoplasma Pneumoniae* Pneumonia in Children” (15). Specifically, corticosteroids were considered for patients presenting with persistent high fever (>38.5°C) combined with radiographic evidence of extensive pulmonary consolidation or pleural effusion, or those exhibiting signs of systemic inflammatory response. While the guideline provides a framework, the final decision involved clinical judgment regarding the need for adjunctive anti-inflammatory therapy.

Based on the pathophysiological principle that SMPP is driven by an exaggerated host immune response peaking within the first 48-72 hours of critical illness, and in accordance with the Evidence-Based Guideline (15), “early corticosteroid use” was defined as the administration of methylprednisolone within 24 hours of admission. This timeframe represents the clinical window available to clinicians to assess disease severity and intervene before the full onset of the cytokine storm. Patients who received corticosteroids after 24 hours of admission were strictly excluded to maintain homogeneity in the intervention timing. For the 101 patients in the early corticosteroid group, the median duration from symptom onset to the first dose of corticosteroid was 7.5 days (IQR: 6.0–9.0 days) in the early corticosteroid group. The distribution of steroid initiation relative to admission time was as follows: <6 hours (*n* = 32, 31.68%), 6–12 hours (*n* = 41, 40.59%), 12–24 hours (*n* = 24, 23.76%), and >24 hours (*n* = 4, 3.96%).

### 2.4 Observation parameters

#### 2.4.1 Pulmonary function improvement

Before and after treatment, a pediatric pulmonary function instrument (MasterScreen PAED, Jaeger, Germany) was used to measure various lung function indicators, including PEF, FEV1, forced vital capacity (FVC), maximum mid-expiratory flow (MMEF), and maximal expiratory flow at 25%, 50%, and 75% of FVC (MEF25, MEF50, MEF75).

Pulmonary function tests (PFTs) were performed by trained pediatric respiratory therapists using a MasterScreen PAED pneumotachometer in accordance with the 2019 American Thoracic Society (ATS)/European Respiratory Society (ERS) standards for spirometry (17). The equipment was calibrated using a 3-liter syringe before each testing session. Room temperature, humidity, and barometric pressure were recorded to standardize volume measurements to body temperature and pressure saturated (BTPS) conditions. Children were instructed using standardized verbal commands and visual incentives. Forced expiratory maneuvers were performed in a seated position with a nose clip. Each child performed at least three acceptable forced expiratory manoeuvres, and the test was terminated when the difference between the two best values of FEV1 and FVC was less than 150 mL (or <5%). The largest FEV1 and FVC values from the acceptable curves were selected for analysis. All tests were administered by certified technicians with >2 years of experience in pediatric pulmonary function testing, and all raw data were reviewed by a senior pediatrician to ensure waveform quality (no artefacts, smooth curve, and complete expiration).

#### 2.4.2 Laboratory finding

① Injury markers: Before and after treatment, an automated biochemical analyzer (AU5800, Beckman Coulter, USA) was used to measure levels of lactate dehydrogenase (LDH), aspartate aminotransferase (AST), and alanine aminotransferase (ALT); ② Inflammatory cytokines: Before and after treatment, an electrochemiluminescence immunoassay analyzer (cobas e 602, Roche Diagnostics, Switzerland) was utilized to assess levels of C-reactive protein (CRP), TNF-α, IL-6, and IFN-γ; ③ Immune function: Before and after treatment, a protein analyzer (IMMAGE 800, Beckman Coulter, USA) was used to measure levels of immunoglobulins (Ig) A, IgG, and IgM.

#### 2.4.3 Clinical symptoms

The time of cough disappearance, time of pulmonary shadows disappearance, time of defervescence, and time of hospitalization were compared between the two groups.

#### 2.4.4 Adverse reactions

The incidence of adverse reactions during treatment, including nausea, vomiting, abdominal pain, rash, hyperglycemia, hypertension, secondary infection, gastrointestinal bleeding, and behavioral effects, was compared between the two groups.

### 2.5 Statistical analysis

All statistical analyses for this study were performed using SPSS software (version 29.0; developed by SPSS Inc., Chicago, IL, USA). Continuous variables were reported as means ± standard deviations (M ± SD) after assessing normality with the Shapiro–Wilk test, whereas categorical variables were presented as frequencies and percentages [*n* (%)]. Group differences were analyzed using independent samples t-tests for continuous variables and chi-square tests for categorical variables. To adjust for potential residual baseline imbalances and to maximize statistical efficiency, an analysis of covariance (ANCOVA) was performed for each continuous outcome measure. In these models, the post-treatment value served as the dependent variable, treatment group (antibiotics alone vs. early corticosteroid use) as the fixed factor, and the corresponding baseline value as a covariate. For time-to-event clinical outcomes, including time of cough disappearance, time of pulmonary shadows disappearance, time of defervescence, time of hospitalization, survival distributions were estimated using the Kaplan–Meier method, and comparisons between groups were conducted using the Log-rank test. A two-tailed p-value < 0.05 was considered statistically significant.

Missing data were minimal (<1% of total observations). For pulmonary function parameters, 2 patients were excluded from the analysis due to unacceptable maneuver quality (failure to meet ATS/ERS repeatability criteria), resulting in complete case analysis for the primary outcomes. No imputation methods were applied, as the missing data were considered missing completely at random (MCAR) and the sample size was sufficient to maintain statistical power.

As this was a retrospective study, formal sample size calculation was not performed prior to data collection. However, a *post-hoc* power analysis was conducted using G*Power software (version 3.1) to confirm the adequacy of the cohort. Based on the observed effect size (Cohen's *d* = 1.25) for the primary outcome of PEF improvement, with an alpha level of 0.05 and the actual sample sizes (*n* = 158 and *n* = 101), the calculated statistical power exceeded 0.99. This indicates that the study was sufficiently powered to detect clinically meaningful differences in pulmonary function between the two groups.

## 3 Results

### 3.1 General data

A total of 259 pediatric patients with SMPP were included in this retrospective analysis: 158 in the antibiotics alone group and 101 in the early corticosteroid use group. As detailed in Table 1, no statistically significant differences were observed between the groups with respect to age, gender distribution, pneumonia type, history of asthma, history of atopic dermatitis, fever duration before admission, peak temperature, SpO₂, WBC, D-dimer, multi-lobar involvement, involvement ≥2/3 of single lobe, pleural effusion, or intensive care unit (ICU) for hospitalization (all *P* > 0.05), indicating that the baseline characteristics were comparable between the two groups.

**Table 1:** Comparison of general data between two groups.

Parameter	Antibiotics alone group (*n* = 158)	Early corticosteroid use group (*n* = 101)	*t/χ^2^*	*P*
Age (years)	9.58 ± 2.24	9.17 ± 2.05	1.476	0.141
Gender [*n* (%)]			0.003	0.958
- Male	73 (46.20%)	47 (46.53%)		
- Female	85 (53.80%)	54 (53.47%)		
Type of pneumonia [*n* (%)]			0.102	0.749
- Bronchopneumonia	83 (52.53%)	51 (50.50%)		
- Lobar pneumonia	75 (47.47%)	50 (49.50%)		
Asthma history [*n* (%)]	11 (6.96%)	8 (7.92%)	0.083	0.773
Atopic dermatitis history [*n* (%)]	14 (8.86%)	10 (9.90%)	0.079	0.778
Fever duration before admission (d)	6.95 ± 2.14	6.69 ± 2.21	0.964	0.336
Peak temperature (°C)	39.13 ± 0.67	39.08 ± 0.72	0.502	0.616
SpO₂ (%)	94.08 ± 2.75	93.62 ± 3.01	1.287	0.199
WBC (× 10^9^/L)	10.96 ± 4.17	11.28 ± 4.56	0.573	0.567
D-dimer (mg/L)	1.26 ± 0.71	1.34 ± 0.79	0.880	0.380
Multi-lobar involvement [*n* (%)]	52 (32.91%)	41 (40.59%)	1.580	0.209
Involvement ≥2/3 of single lobe [*n* (%)]	38 (24.05%)	32 (31.68%)	1.820	0.177
Pleural effusion [*n* (%)]	22 (13.92%)	19 (18.81%)	1.105	0.293
ICU for hospitalization [*n* (%)]	6 (3.80%)	5 (4.95%)	0.018	0.894

SpO₂, oxygen saturation; WBC, white blood cell count; ICU, intensive care unit.

### 3.2 Pulmonary function improvement

**Figure 1:**
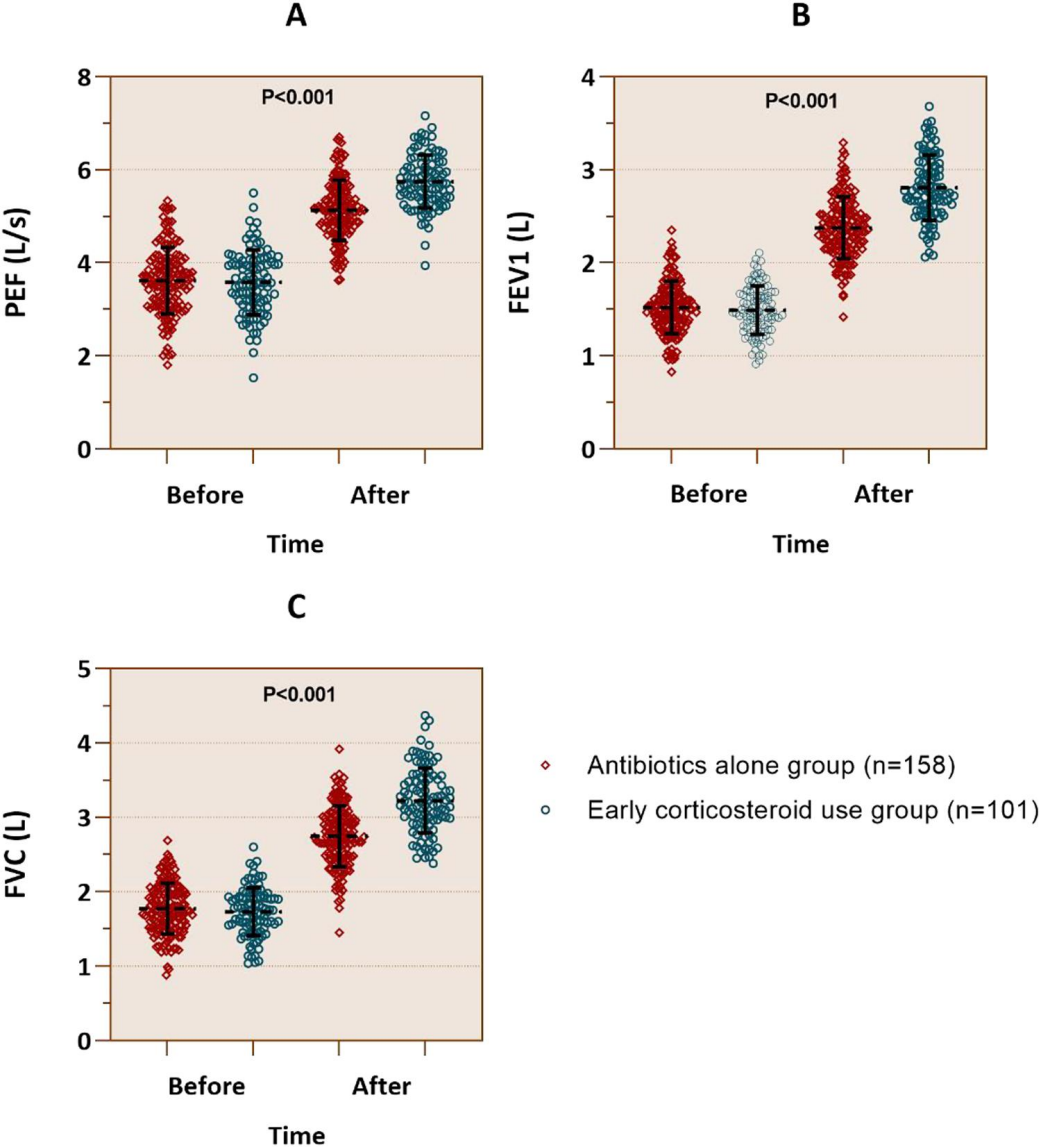
Comparison of ventilation function indicators between the antibiotics alone group (*n* = 158) and the early corticosteroid use group (*n* = 101). **(A)** Peak expiratory flow (PEF); **(B)** Forced expiratory volume in the first second (FEV1); **(C)** Forced vital capacity (FVC). Pre-treatment values are shown on the left and post-treatment values on the right for each group. Data are presented as means ± SD. *P-values* were derived from ANCOVA models adjusting for baseline values.

As presented in Figure 1, no significant differences were observed in baseline ventilation function indicators, including PEF, FEV1, and FVC, between the two groups prior to treatment (all *P* > 0.05). After treatment, however, patients in the early corticosteroid use group had significantly higher PEF (*F* = 33.137, *P* < 0.001, ges = 0.055), FEV1 (*F* = 65.247, *P* < 0.001, ges = 0.117), and FVC (*F* = 53.581, *P* < 0.001, ges = 0.102) than those in the antibiotics alone group.

For small airway function indicators (Table 2), no significant between-group differences were observed before treatment (all *P* > 0.05). After treatment, the early corticosteroid use group demonstrated significantly greater improvements in MEF25 (*F* = 5.521, *P* = 0.020, ges = 0.010), MEF50 (*F* = 5.133, *P* = 0.024, ges = 0.010), MEF75 (*F* = 5.887, *P* = 0.016, ges = 0.010), and MMEF (*F* = 7.759, *P* = 0.006, ges = 0.014) compared to the antibiotics alone group.

**Table 2:** Comparison of small airway function indicators between two groups (L/s).

Parameter	Antibiotics alone group (*n* = 158)	Early corticosteroid use group (*n* = 101)	*F*	*P*	ges
MEF25			5.521	0.020	0.010
- Before treatment	1.32 ± 0.41	1.28 ± 0.39			
- After treatment	2.56 ± 0.48	2.71 ± 0.52			
MEF50			5.133	0.024	0.010
- Before treatment	2.08 ± 0.57	2.03 ± 0.54			
- After treatment	3.67 ± 0.63	3.86 ± 0.68			
MEF75			5.887	0.016	0.010
- Before treatment	3.14 ± 0.72	3.09 ± 0.69			
- After treatment	4.99 ± 0.76	5.24 ± 0.81			
MMEF			7.757	0.006	0.014
- Before treatment	1.84 ± 0.49	1.79 ± 0.47			
- After treatment	3.36 ± 0.55	3.57 ± 0.61			

MEF25, maximal expiratory flow at 25% of forced vital capacity; MEF50, maximal expiratory flow at 50% of forced vital capacity; MEF75, maximal expiratory flow at 75% of forced vital capacity; MMEF, maximal mid-expiratory flow.

### 3.3 Laboratory finding

Regarding injury markers (Table 3), baseline levels of LDH, AST, and ALT were comparable between the two groups (all *P* > 0.05). After treatment, patients receiving early corticosteroids showed significantly lower levels of LDH (*F* = 13.520, *P* < 0.001, ges = 0.025) compared to those receiving antibiotics alone.

**Table 3:** Comparison of injury markers between two groups (U/L).

Parameter	Antibiotics alone group (*n* = 158)	Early corticosteroid use group (*n* = 101)	*F*	*P*	ges
LDH			13.520	<0.001	0.025
- Before treatment	635.48 ± 152.36	628.71 ± 148.95			
- After treatment	492.36 ± 118.74	398.62 ± 105.43			
AST			1.345	0.247	0.003
- Before treatment	48.27 ± 15.73	47.15 ± 15.29			
- After treatment	32.41 ± 10.25	28.53 ± 8.96			
ALT			0.990	0.321	0.002
- Before treatment	32.97 ± 8.34	31.96 ± 7.82			
- After treatment	22.53 ± 6.61	20.18 ± 5.74			

LDH, lactate dehydrogenase; AST, aspartate aminotransferase; ALT, alanine aminotransferase.

For inflammatory cytokines (Table 4), no significant differences were detected in baseline CRP, TNF-α, IL-6, or IFN-γ levels between groups (all *P* > 0.05). Post-treatment, the early corticosteroid use group exhibited notably lower levels of CRP (*F* = 5.171, *P* = 0.024, ges = 0.010) and IL-6 (*F* = 24.514, *P* < 0.001, ges = 0.041) compared to the antibiotics alone group.

**Table 4:** Comparison of inflammatory cytokines between two groups.

Parameter	Antibiotics alone group (*n* = 158)	Early corticosteroid use group (*n* = 101)	*F*	*P*	ges
CRP (mg/L)			5.171	0.024	0.010
- Before treatment	45.83 ± 12.67	44.91 ± 12.35			
- After treatment	12.35 ± 3.28	7.62 ± 2.14			
TNF-α (pg/mL)			0.165	0.685	<0.001
- Before treatment	28.46 ± 6.73	27.89 ± 6.58			
- After treatment	10.43 ± 3.41	9.47 ± 2.36			
IL-6 (pg/mL)			24.514	<0.001	0.041
- Before treatment	52.37 ± 14.82	51.64 ± 14.39			
- After treatment	24.51 ± 6.75	14.28 ± 4.53			
IFN-γ (pg/mL)			0.973	0.325	0.002
- Before treatment	42.68 ± 11.24	41.95 ± 10.98			
- After treatment	26.71 ± 6.52	24.39 ± 5.16			

CRP, C-reactive protein; TNF-α, tumor necrosis factor-alpha; IL-6, interleukin-6; IFN-γ, interferon-gamma.

In terms of immune function (Table 5), baseline immunoglobulin levels (IgA, IgG, IgM) were similar between the two groups (all *P* > 0.05). After treatment, the early corticosteroid use group demonstrated notably higher levels of IgA (*F* = 5.894, *P* = 0.016, ges = 0.013), IgG (*F* = 5.276, *P* = 0.022, ges = 0.010), and IgM (*F* = 4.973, *P* = 0.027, ges = 0.010) compared to the antibiotics alone group.

**Table 5:** Comparison of immune function between two groups (g/L).

Parameter	Antibiotics alone group (*n* = 158)	Early corticosteroid use group (*n* = 101)	*F*	*P*	ges
IgA			5.894	0.016	0.013
- Before treatment	0.84 ± 0.21	0.82 ± 0.23			
- After treatment	1.45 ± 0.33	1.57 ± 0.35			
IgG			5.276	0.022	0.010
- Before treatment	7.92 ± 1.53	7.86 ± 1.49			
- After treatment	12.09 ± 1.84	12.73 ± 1.96			
IgM			4.973	0.027	0.010
- Before treatment	1.38 ± 0.42	1.35 ± 0.41			
- After treatment	1.53 ± 0.28	1.64 ± 0.32			

Ig, immunoglobulin.

### 3.4 Clinical symptoms

**Figure 2:**
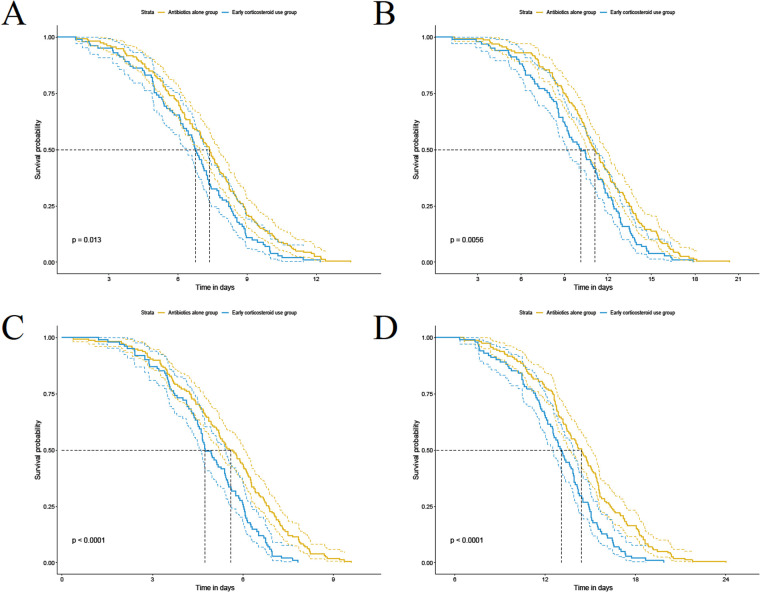
Kaplan–Meier curves comparing the cumulative probability of clinical event occurrence between the antibiotics alone group (*n* = 158) and the early corticosteroid use group (*n* = 101). **(A)** Time of cough disappearance; **(B)** Time of pulmonary shadows disappearance; **(C)** Time of defervescence; **(D)** Time of hospitalization. *P-values* were calculated using the log-rank test. Vertical dashed lines indicate median event times for each group.

Kaplan–Meier analysis was performed to compare the clinical recovery trajectories between the two groups (Figure 2). As shown in Figure 2, the early corticosteroid use group exhibited a significantly faster rate of defervescence compared to the antibiotics alone group (median time: 4.0 days vs. 5.0 days; Log-rank *χ^2^* = 9.92, *P* < 0.001). Similarly, the cumulative probability of hospital discharge was markedly higher in the corticosteroid group, with a significantly shortened median length of stay (12.0 days vs. 14.0 days; Log-rank *χ^2^* = 13.36, *P* < 0.001). Consistent with these findings, the time to cough disappearance and pulmonary shadow resolution were also notably shorter in the corticosteroid group (both *P* < 0.05). Mean values and standard deviations for these clinical parameters are presented in Table 6.

**Table 6:** Comparison of clinical symptoms between two groups (d).

Parameter	Antibiotics alone group (*n* = 158)	Early corticosteroid use group (*n* = 101)	*t*	*P*
Time of cough disappearance	7.28 ± 2.35	6.64 ± 2.17	2.178	0.030
Time of pulmonary shadows disappearance	11.16 ± 3.37	9.98 ± 3.29	2.782	0.006
Time of defervescence	5.49 ± 1.83	4.84 ± 1.46	3.150	0.002
Time of hospitalization	14.34 ± 3.26	12.91 ± 2.74	3.657	< 0.001

### 3.5 Adverse reactions

The adverse events occurring during treatment are summarized in Table 7. The overall incidence of adverse reactions was 11.39% in the antibiotics alone group and 14.85% in the early corticosteroid use group, with no statistically significant difference observed between the two groups (*P* = 0.511).

**Table 7:** Comparison of adverse reactions incidence between two groups [*n* (%)].

Parameter	Antibiotics alone group (*n* = 158)	Early corticosteroid use group (*n* = 101)	*χ^2^*	*P*
Total incidence	19 (11.39%)	15 (14.85%)	0.432	0.511
- Nausea	9 (5.70%)	7 (6.93%)		
- Vomiting	5 (3.16%)	4 (3.96%)		
- Abdominal pain	3 (1.90%)	2 (1.98%)		
- Rash	1 (0.63%)	1 (0.99%)		
- Hyperglycemia	0 (0.00%)	3 (2.97%)		
- Hypertension	0 (0.00%)	1 (0.99%)		
- Secondary infection	2 (1.27%)	3 (2.97%)		
- Gastrointestinal bleeding	0 (0.00%)	0 (0.00%)		
- Behavioral effects	1 (0.63%)	3 (2.97%)		

## 4 Discussion

This retrospective study investigated the impact of early corticosteroid intervention on the recovery of children with SMPP. The findings indicate that the addition of corticosteroids to standard antibiotic therapy was associated with more favorable short-term outcomes compared to antibiotics alone in this retrospective cohort. Children who received early corticosteroids demonstrated greater short-term improvement in both large airway and small airway function parameters measured at the completion of the acute treatment phase. This improvement in pulmonary outcomes was accompanied by a more pronounced reduction in markers of tissue injury and a greater suppression of pro-inflammatory cytokines. Furthermore, the corticosteroid group experienced quicker resolution of clinical symptoms, including fever and cough, and a shorter hospital stay, all without an apparent increase in treatment-related adverse events.

The observed improvements in pulmonary function are a key finding of this study. While both treatment groups showed expected recovery following the acute infection, the degree of improvement in parameters such as PEF, FEV1, and FVC was more substantial in the group receiving early corticosteroids. This pattern extended to the small airways, where indicators like MMEF and maximal expiratory flow at various lung volumes also showed greater recovery. The pathogenesis of SMPP involves not only direct pathogen injury but also a robust host immune response that can lead to airway inflammation, edema, and subsequent remodeling. This process can result in persistent airflow limitation even after the infection is cleared. By suppressing this hyperinflammatory state, corticosteroids may attenuate acute airway damage and thereby facilitate more complete functional recovery (18, 19). These findings align with research by Luo et al., who reported that children with SMPP treated with corticosteroids had improved forced expiratory flow compared to those on antibiotics alone, suggesting a protective effect on lung function (20). Similarly, a study by You et al. highlighted that delayed recovery of lung function in some children with refractory MPP could be ameliorated by timely anti-inflammatory therapy (21).

The laboratory findings provide insights into the potential mechanisms underlying these clinical benefits. Children in the early corticosteroid group exhibited lower post-treatment levels of lactate dehydrogenase, a marker often correlated with the extent of pulmonary inflammation and tissue damage in MPP (22). This suggests that early immune modulation may help control the inflammatory process, thereby reducing collateral injury to the lung parenchyma. This is further supported by the more pronounced decrease in inflammatory cytokines, particularly CRP and IL-6, in the corticosteroid group (23). IL-6 is a central driver of the acute phase response and fever in MPP, and its effective suppression likely contributes to the faster defervescence and clinical improvement observed. By dampening this cascade, corticosteroids may interrupt the cycle of inflammation and tissue destruction (24). These results are consistent with the work of Fang et al., who reported that adjuvant corticosteroids in children with SMPP were associated with a more rapid decline in inflammatory markers and better clinical outcomes (25).

An intriguing finding of this study was the observation of significantly higher post-treatment IgA, IgG, and IgM levels in the early corticosteroid group compared to the antibiotics alone group. At first glance, this appears counterintuitive, as systemic corticosteroids are generally recognized to suppress adaptive immune responses and antibody production (26, 27). However, in the context of severe inflammation, excessively high levels of pro-inflammatory cytokines (such as IL-6) can lead to immune dysregulation or “immune paralysis,” potentially impairing effective humoral responses (18, 24). We hypothesize that by rapidly curtailing this hyperinflammatory milieu, early corticosteroid intervention may have indirectly allowed for a more orderly immune reconstitution (28). Nonetheless, this interpretation remains highly speculative. An alternative and perhaps more parsimonious explanation is that the elevated immunoglobulin levels simply reflect standard recovery-phase kinetics. Given that the corticosteroid group exhibited faster defervescence and shorter hospital stays, these patients may have entered the convalescent phase earlier, during which physiologic rebound or normalization of immunoglobulin synthesis naturally occurs. Without serial measurements of specific MP antibodies or cellular phenotyping (e.g., T and B cell subsets), we cannot definitively distinguish between these possibilities. Therefore, this finding should be viewed as a descriptive observation rather than a proven mechanistic effect.

From a clinical perspective, the faster resolution of fever, cough, and pulmonary infiltrates in the corticosteroid group translates directly into tangible patient benefits, including a shorter hospital stay. This not only reduces the burden on the healthcare system but also minimizes the disruption to the child's and family's life. The quicker defervescence is likely a direct consequence of the effective suppression of pyrogenic cytokines like interleukin-6, while the faster clearance of pulmonary shadows and cough may be attributed to the reduction in airway inflammation and edema, promoting better mucociliary clearance (29). Importantly, combination therapy did not result in a higher incidence of adverse reactions, suggesting that this short-course early intervention strategy is well tolerated in this population. This safety profile is crucial when considering the adoption of such an approach in clinical practice (30, 31).

This investigation has several limitations that warrant consideration. First, the retrospective, non-randomized design inherently carries a risk of selection bias and confounding by indication. Although we applied multivariable ANCOVA adjustment to reduce observed confounding, we acknowledge that unmeasured confounders (such as clinician gestalt, subtle physical examination findings, or family preferences) may still influence the results. Second, we did not perform propensity score matching (PSM) because the relatively small corticosteroid group (*n* = 101) would have been substantially reduced by 1:1 matching, leading to a loss of statistical power that could obscure potentially important differences. Moreover, our baseline comparisons showed good balance on measured severity indicators. Nonetheless, the absence of PSM remains a limitation, and readers should interpret the findings with appropriate caution. Third, we did not perform molecular testing for macrolide resistance (e.g., 23S rRNA gene mutations). Although the high prevalence of macrolide-resistant MP in China (often exceeding 80%) suggests that the majority of our cohort likely harbored resistant strains, the lack of specific resistance data limits our ability to correlate treatment response with microbial genotype. Fourth, the interpretation of the immunoglobulin findings is limited by the lack of mechanistic data. While we observed higher post-treatment levels in the steroid group, we did not measure specific MP antibodies or assess lymphocyte subsets and cytokine profiles beyond CRP and IL-6. Consequently, we cannot determine whether this represents a true immunomodulatory effect of steroids or merely a nonspecific reflection of faster clinical recovery. Future studies incorporating flow cytometry and antigen-specific antibody assays are needed to elucidate this phenomenon. Fifth, our safety analysis was limited by the retrospective design. We likely underestimated the incidence of subjective or less documented adverse events, such as mild behavioral changes, transient hyperglycemia, or asymptomatic secondary infections. Additionally, the 7-day follow-up period precluded assessment of rare or long-term complications associated with corticosteroid use. Prospective registries with standardized adverse event reporting are needed to fully characterize the safety profile of early corticosteroid therapy in SMPP. Future research should prioritize prospective, randomized controlled trials with larger, multi-center cohorts to confirm these findings. Long-term follow-up studies incorporating serial pulmonary function tests are essential to determine if early corticosteroid intervention can prevent the chronic respiratory sequelae, such as persistent airway hyperreactivity or obstructive defects, that are sometimes seen after SMPP. Additionally, studies exploring optimal dosing regimens and identifying specific patient subgroups most likely to benefit from this therapy would help refine clinical guidelines.

## 5 Conclusion

In summary, this retrospective study suggests that, in pediatric patients with SMPP, early introduction of corticosteroids alongside standard antibiotic therapy was associated with greater short-term improvement in both large and small airway function, more pronounced reductions in systemic inflammation and markers of tissue injury, and faster clinical symptom resolution, leading to a shorter hospital stay, without an observed increase in acute adverse events. However, due to the observational design, these findings should be interpreted as associations rather than evidence of causality. They suggest that early corticosteroid therapy may warrant further investigation in prospective, randomized controlled trials to definitively establish its efficacy and safety in this population.

## Data Availability

The raw data supporting the conclusions of this article will be made available by the authors, without undue reservation.
